# Attenuated mutant strain of Salmonella Typhimurium lacking the ZnuABC transporter contrasts tumor growth promoting anti-cancer immune response

**DOI:** 10.18632/oncotarget.3893

**Published:** 2015-05-07

**Authors:** Barbara Chirullo, Serena Ammendola, Leonardo Leonardi, Roberto Falcini, Paola Petrucci, Claudia Pistoia, Silvia Vendetti, Andrea Battistoni, Paolo Pasquali

**Affiliations:** ^1^ Department of Veterinary Public Health and Food Safety, Istituto Superiore di Sanità, Rome 00161, Italy; ^2^ Department of Biology, University of Rome Tor Vergata, Rome 00133, Italy; ^3^ Università degli Studi di Perugia, Department of Veterinary Medicine, Perugia 06126, Italy; ^4^ Veterinary Clinic, Rieti 02043, Italy; ^5^ Department of Infectious, Parasitic and Immune-Mediated Diseases, Istituto Superiore di Sanità, Rome 00161, Italy

**Keywords:** bacterial therapy, cancer therapy, antitumor efficacy, attenuated-Salmonella, immune response

## Abstract

*Salmonella* Typhimurium has been shown to be highly effective as antitumor agent. The aim of this study was to investigate the tumor targeting efficacy and the mechanism of action of a specific attenuated mutant strain of *Salmonella* Typhimurium (STM) devoid of the whole operon coding for the high-affinity zinc transporter ZnuABC, which is required for bacterial growth in environments poor in zinc and for conferring full virulence to different Gram-negative pathogens.

We showed that STM is able to penetrate and replicate into tumor cells in *in vitro* and *in vivo* models. The subcutaneous administration of STM in mammary adenocarcinoma mouse model led to both reduction of tumor growth and increase in life expectancy of STM treated mice. Moreover, investigating the potential mechanism behind the favorable clinical outcomes, we provide evidence that STM stimulates a potent inflammatory response and a specific immune pattern, recruiting a large number of innate and adaptive immune cells capable to contrast the immunosuppressive environment generated by tumors.

## INTRODUCTION

The use of bacteria in cancer therapy has been studied for more than a century. Since the 19^th^ century, there are many evidences showing that solid tumors may undergo regression after bacterial infection [1]. This approach has lately received a renewed interest because bacteria have shown some selective replication and preferential accumulation in tumor areas. Recent advances in tumor targeting with attenuated *Salmonella* enterica serovar Typhimurium (S. Typhimurium), a facultative intracellular bacterium capable to grow anaerobically, have highlighted the great potentiality of this pathogen for the development of new strategies for cancer therapy, directed on both primary [2–10] and metastatic tumors [11–16]. Attenuation of virulence, besides its advantages in treatment safety, is crucial for the development of strains able to elicit an appropriate profile of the immune response. Most of the current attenuated-*Salmonella* strains investigated as anti-cancer tools have been obtained by inactivation of genes coding for proteins involved in metabolic pathways. Conversely, we have selected an attenuated mutant strain of S. Typhimurium, henceforth defined STM, characterized by a deletion of the whole *znuABC* operon, coding for the high-affinity zinc transporter [17]. The ZnuABC transporter confers a selective advantage for the growth of bacteria in environments poor in zinc and for virulence of different gram-negative pathogens [18]. This strain has been extensively tested for vaccination purposes in different animal species (e.g. mice, pigs) demonstrating that it is able to elicit effective protection against systemic and enteric salmonellosis [19, 20]. Therefore, the impairment of the high-affinity zinc transport system represents a new and valid approach to attenuate bacteria without altering their capability to induce a suitable immune response [17, 19, 21–23].

In the present study, we evaluate the antitumor effect of STM in a mammary adenocarcinoma mouse model and the involvement of immune response, to pose the basis to understand the STM-mechanism of action since the general understanding of attenuated-Salmonella anti-tumor mechanistic explanation is still largely undisclosed. Our findings provide evidence that the administration of STM induces statistically significant results for both reduction of the tumor growth and increase in the average life expectancy of tumor-bearing immunocompetent mice. Moreover, we ascertained the engagement of an antitumor immune pattern able to induce favorable clinical outcomes.

## RESULTS

### STM penetrates into tumor cells and inhibits their proliferation *in vitro*

**Figure 1:**
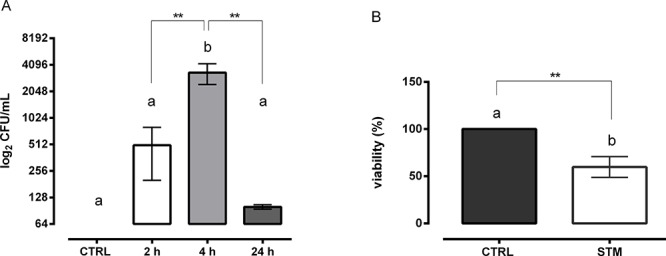
STM colonizes mouse mammary adenocarcinoma cells and reduces their proliferation **A.** Intracellular colonization of STM in 4T1 tumor cells at 2, 4 and 24 h post-treatment (Mann-Whitney unpaired *t*-test). **B.** Viability of 4T1 tumor cells 24 h after STM treatment (Mann-Whitney unpaired *t*-test).

We performed *in vitro* experiments using the mouse mammary adenocarcinoma 4T1 cell line. We observed that STM was able to efficiently colonize 4T1 tumor cells and that the number of intracellular bacteria reached a maximum at 4 h (Figure 1A). In order to assess if STM is able to penetrate into cells of different embryologic nature and animal species, we used the TC1 cell line, a mouse lung tumor cell line co-transformed with human papillomavirus 16 E6/E7 and c-Ha-Ras, and the SiHa cell line, a human tumor cell line (grade II of human squamous carcinoma cell of cervix). As shown in Supplementary Figure S1, STM was able to penetrate in both tumor cell lines.

A significant reduction in the proliferation of 4T1 cells was also observed. The results indicated that STM induced a direct action on the tumor cell viability leading to a 40% reduction in the number of viable cells as compared to control cells at 24 h post-treatment, as measured by MTT assay (*P* = 0.002, Student's *T* test; Figure 1B). Overall, *in vitro* studies showed that STM is able to penetrate into the tumor cell lines and to significantly reduce their viability.

### STM induces a significant reduction of the tumor growth and prolongs survival when co-administrated with 4T1 tumor cells

**Figure 2:**
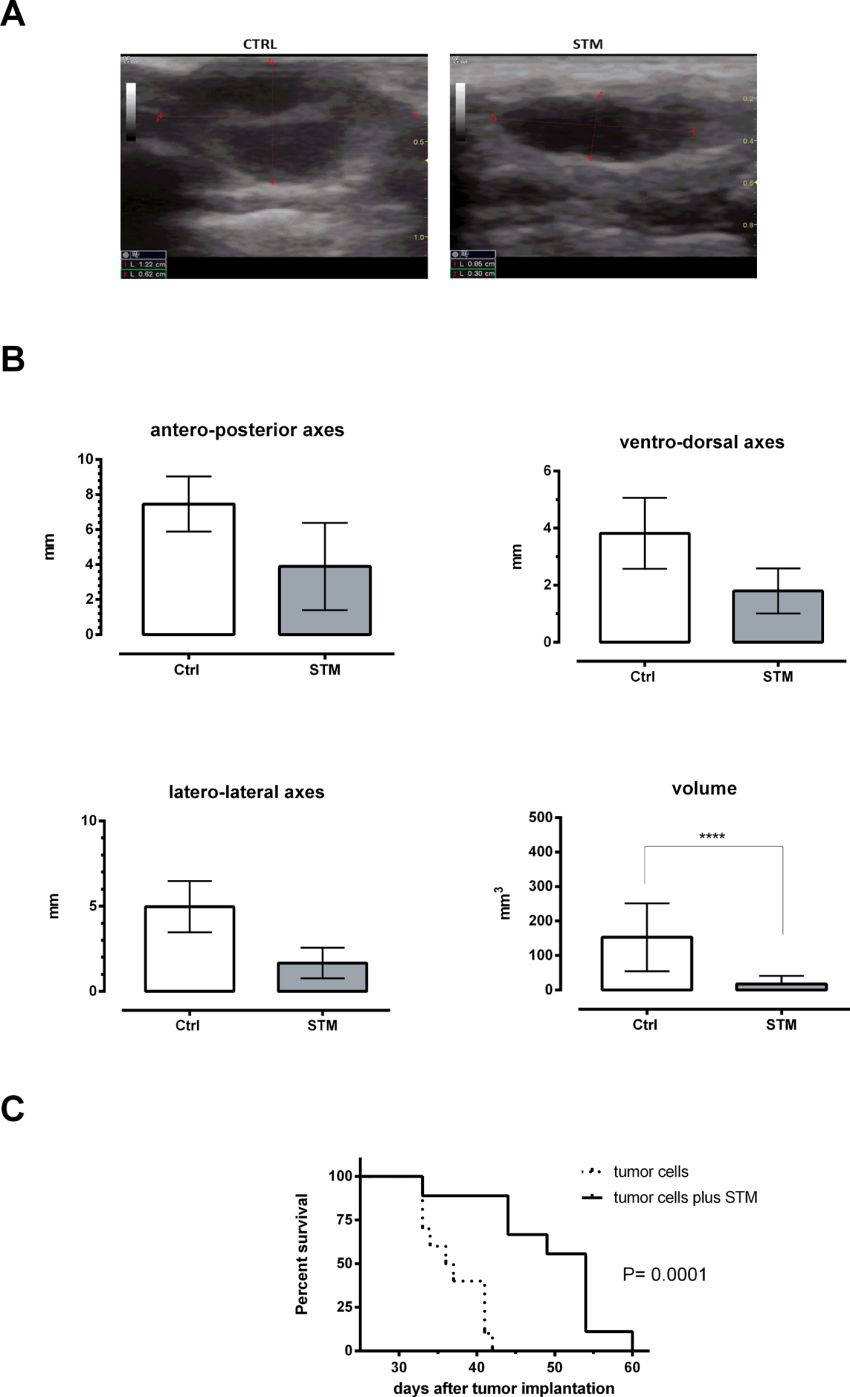
STM when co-administered with tumor cells determines a significant reduction of the tumor masses and a significant increase of average life expectancy in Balb/C mice **A.** Picture from ultrasound analysis. **Control group:** infiltrative hypoechogenic nodular mass infiltrating the peritoneal fascia and separated by hypoechogenic septum. The infiltration of the mass involves also surrounding soft tissues. **STM group:** the mass appear uniformly nodular and hypoechogenic not infiltrative of the peritoneal fascia. The nodule appears well circumscribed and not infiltrative in the surrounding tissues of the breast. The pictures refer to one out of ten mice each group with similar results. **B.** Dimensions of principal axes and total volume of tumor masses, assessed 14 days after administration of 10^3^ CFU of STM and 10^4^ tumor cells by ultrasound analysis (Student's *t*-test). **C.** Mortality curve conducted on two groups of 10 mice each: dashed line identifies the group administered with tumor cells, solid line the group co-administered with tumor cells plus STM (log-rank mantel-cox test).

A first experiment to evaluate the ability of STM to control tumor growth *in vivo* was conducted on immunocompetent Balb/c mice. A first group was subcutaneously (SC) inoculated with 4T1 tumor cells, thus reproducing a well-established animal model mimicking a stage IV human breast cancer [24]. A second group was SC inoculated with 10^3^ CFU of STM in co-administration with 5 × 10^4^ 4T1 tumor cells. Starting 10 days after the first inoculation this group of animals was weekly SC inoculated with the same dose of bacteria. The group 3 was only SC inoculated with 10^3^ CFU of STM. Two weeks after the first co-administration, the total volume of tumor mass was assessed measuring the length of the principal axes by ultrasound analysis (Figure 2A). The results showed that the co-administration of tumor cells and STM leads to a significant reduction of the tumor masses in mice (*P* = 0.0001; Figure 2B). Moreover, differences in tumor growth kinetics over time were paralleled by differences in survival rates, demonstrating that the STM co-administration significantly increases the average life expectancy of the treated-animals compared to the untreated-control group (*P* = 0.0001, Figure 2C). All tumor-free animals treated with STM remained alive throughout the experiment (data not shown).

### STM has selective tropism for tumor mass

To evaluate the bacteria colonization in the tumor mass compared with that in the spleen, some mice were sacrificed and tumors and spleens were removed. Approximately 12 CFU/mg and 155 CFU/mg were found in spleen of tumor-free and tumor-bearing STM-treated mice, respectively. Approximately 3.3 × 10^5^ CFU/mg in tumor mass of STM-treated group were found (5 animals per group, *P* < 0.01). These results suggest a high tropism of STM for tumor mass and, additionally, the higher capability of STM to colonize the spleen of tumor-bearing mice compared to that of free-tumor STM-treated mice (Supplementary Figure S2A). Moreover, the weight of the tumor masses in mice treated with STM were measured and compared to the untreated tumor-bearing mice, showing a reduction of the tumor weight of ~6.5 fold with respect to the untreated control (*P* < 0.0001; Supplementary Figure S2C). The spleens weight was increased in both STM-treated and untreated mice compared the control groups (Supplementary Figure S2B).

### Therapeutic administration of STM significantly reduces tumor growth and prolongs survival

**Figure 3:**
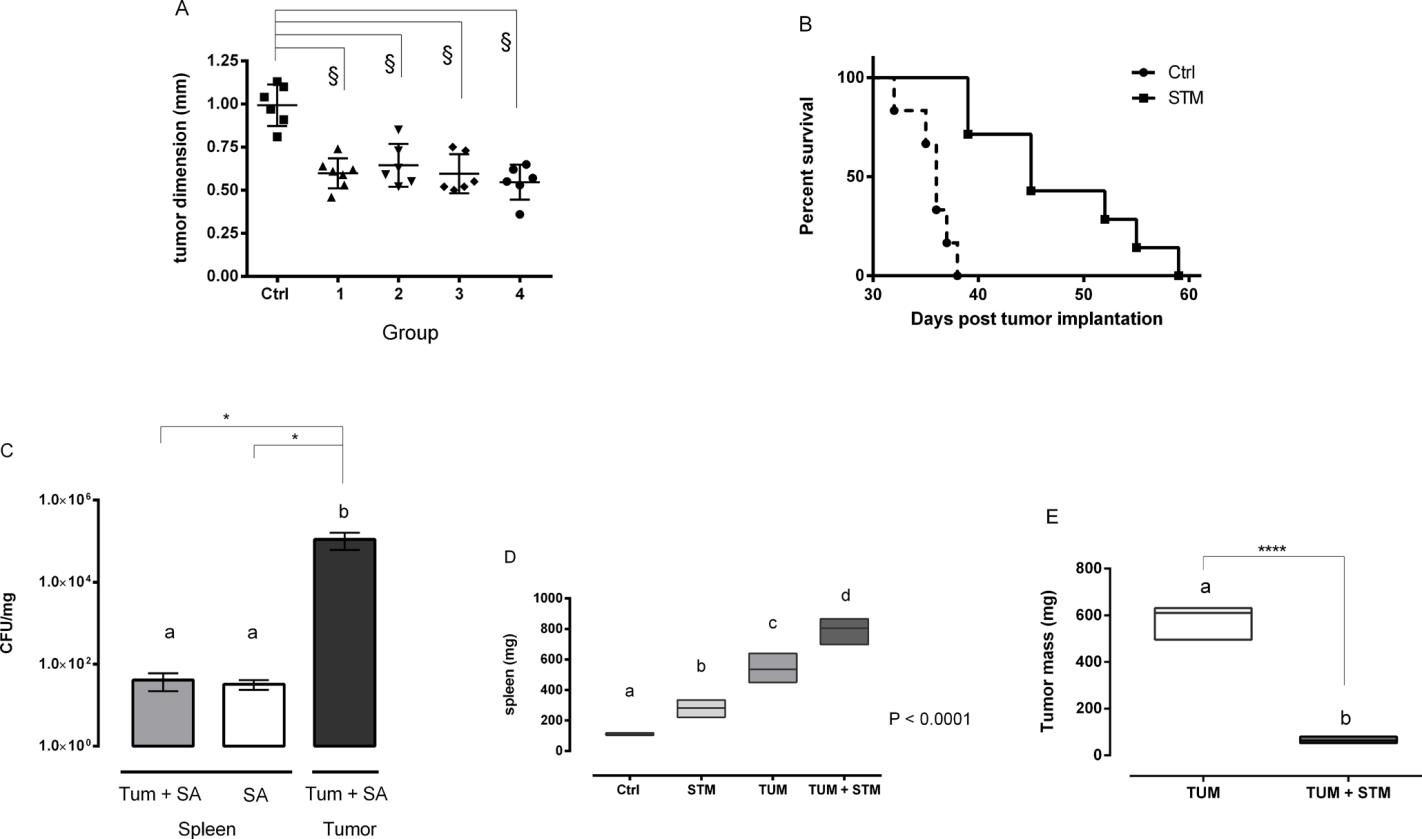
STM reduces tumor growth when administered three or five days after tumor implantation **A.** Dimensions of the tumor masses assessed 14 days PTI and treated with different STM-regimens (§*P* < 0.0001; Dunnett's multiple comparisons Anova test). **B.** Mortality curve conducted on group 1 and control: dashed line identifies the group administered with tumor cells, solid line the one with tumor cells plus STM (log-rank mantel-cox test). **C.** Tumor-bearing and free-tumor mice treated with STM were sacrificed at days 30 PTI and the number of bacteria in tumor and spleen was determined. Graphs show mean of CFU per mg of tissue (Mann-Whitney unpaired *t*-test). **D** and **E.** Weight of spleens and tumor masses of STM-treated and untreated tumor-bearing mice (6 animals per group, one-way Anova Turkey's multiple comparisons and *t*-test analysis).

To mimic more realistic conditions of tumor treatment, a subsequent experiment involved the administration of STM in Balb/c mice after three or five days post-tumor implantation (PTI). Mice were SC inoculated with 5 × 10^4^ 4T1 tumor cells and subsequently with STM with different regimens. The group 1 was SC inoculated with 10^3^ CFU of STM at day 3 PTI, three times in the first week, twice in the second and third week and once in the fourth one. The group 3 was inoculated with 2 × 10^3^ CFU of STM, at day 5 PTI, with the same regimen of the group 1. The groups 2 and 4, instead, were inoculated weekly (both starting at day 3 PTI) with 10^3^ and 5 × 10^3^ CFU of STM, respectively. The group 5 was only SC inoculated, with the same regimen of the groups 1 and 3, with 10^3^ CFU of STM (STM-control group). A tumor bearing control group was inoculated only with saline. This therapeutic protocol showed that STM is able to significantly reduce tumor growth (*P* < 0.0001; Figure 3A) and to increase the average life expectancy of mice (*P* < 0.0001; Figure 3B) also when administered PTI, confirming and extending the previously obtained results.

Moreover, some tumor-bearing mice of the group 1 and STM-control group were sacrificed at day 10 and 30 PTI and tumors and spleens were removed to measure STM-colonization. A high number of STM was recovered from tumors at day 10 (Supplementary Figure S3), increasing at day 30 (Figure 3C). STM was also found in spleens, in both tumor-bearing and tumor-free mice, although in numbers markedly reduced as compared with those found in tumors (Figure 3C). These results demonstrate that STM effectively replicates in the tumor site. Additionally, there was a significant increase (~7-fold) in the spleen weight from mice that received the STM compared to untreated mice, and 2.5-fold compared with STM-treated tumor-free mice (*p* < 0.0001, Figure 3D). At the same time, tumor-bearing STM-treated mice showed a reduction of the tumor weight of about 9-fold with respect to the untreated control (*P* < 0.0001; Figure 3E).

It is known that tumors generated by SC inoculation of 4T1 cells disseminate and induce lung metastases [25]. Hence, to examine whether the reduction of tumor growth and the increased survival in animals treated with STM were correlated to a lower capacity of 4T1 cells to develop metastasis, we investigated the presence of lung tumor nodules in tumor-bearing mice STM-treated compared with the untreated control group. As shown in Supplementary Figure S4, representative of one animal out of 5 per group, there was a remarkable decrease in the incidence of the metastases in STM-treated mice compared to control group.

**Figure 4:**
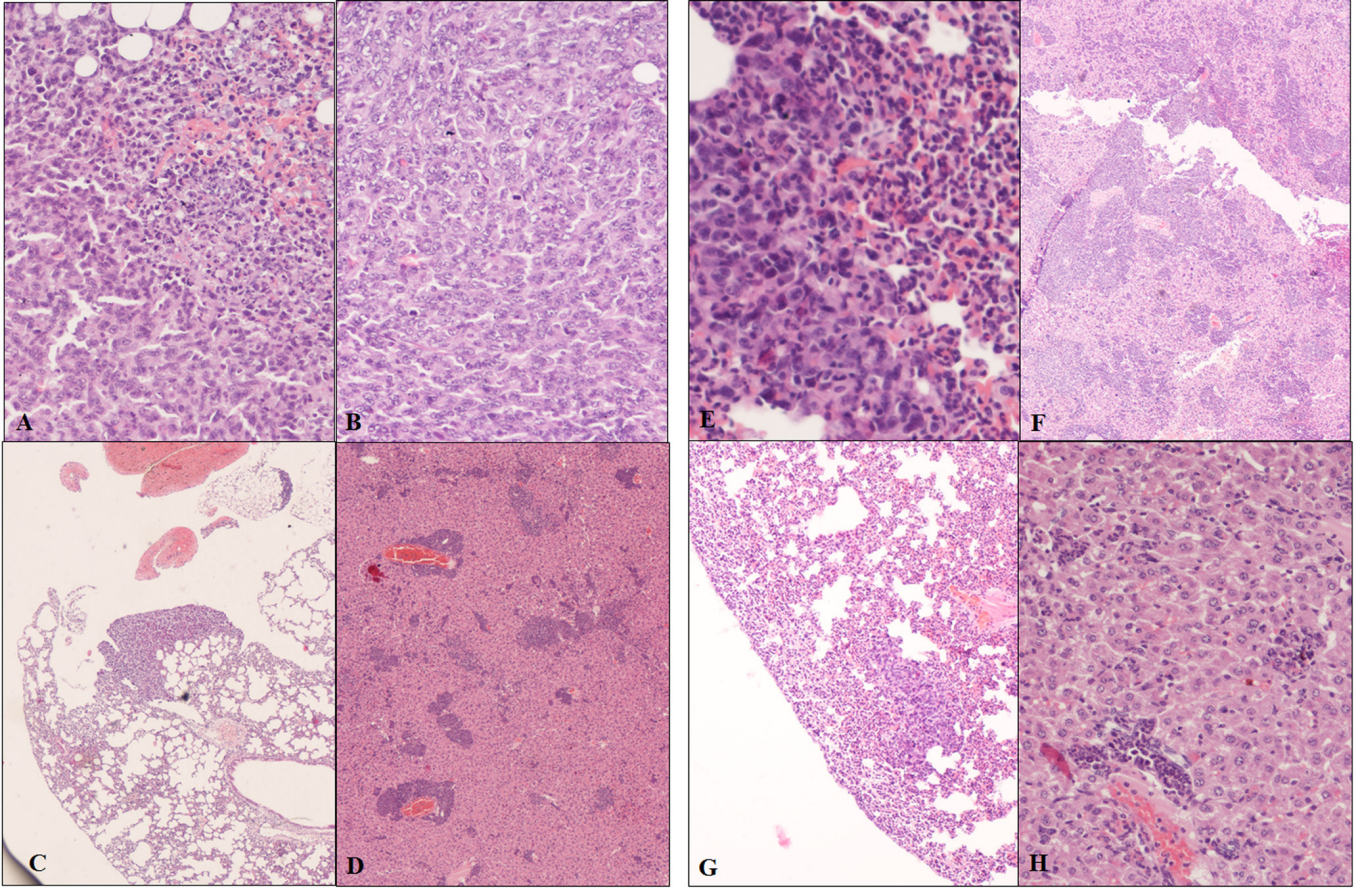
Histopathological sections from primary and metastatic tumors of STM-treated and untreated bearing-tumor mice **Panels A, B, C, D**: Untreated bearing-tumor group. **A.** Primary adenocarcinoma. Neoplastic cells irregularly packed, sometimes with a lobular-like pattern, with marked anisocytosis and anisokaryosis, basophilic nuclei, prominent nucleoli and frequent mitotic figures. Peripheral hemorrhage is present, associated with a moderate inflammatory infiltrate consisting primarily of polymorphonuclear cells and lesser numbers of plasma cells. Hematoxylin-eosin, magnification 20×. **B.** Primary adenocarcinoma. Neoplastic cells irregularly packed, sometimes with a lobular-like pattern, with marked anisocytosis and anisokaryosis, basophilic nuclei, prominent nucleoli and frequent mitotic figures and infiltration of polymorphonuclear leukocytes and plasma cells. Hematoxylin-eosin, magnification 40×. **C.** Adenocarcinoma, lung metastasis. Metastatic nodule, with neoplastic cells that maintain the same morphological features of the primary tumor, associated with an infiltration of polymorphonuclear leukocytes and plasma cells. Hematoxylin-eosin, magnification 20×. **D.** Adenocarcinoma, liver metastasis. Disseminated nodules in the liver. Islands of of neoplastic tissue are present throughout the liver parenchyma. The low grade inflammatory infiltrate is characterized by polymorphonuclear leukocytes and plasma cells. Hematoxylin-eosin, magnification 10×. **Panels E, F, H, G**: STM treated bearing-tumor group. **E.** Primary adenocarcinoma. Neoplastic cells irregularly packed, sometimes with lobular-like pattern, with marked anisocytosis and anisokaryosis, basophilic nuclei, prominent nucleoli. Right side: massive infiltration of neutrophils and eosinophils. Hematoxylin-eosin, magnification 20×. **F.** Primary adenocarcinoma. Large numbers of inflammatory cells mixed with tumor cells. Areas of necrosis are also present. Hematoxylin-eosin, magnification 10×. **G.** Adenocarcinoma, lung metastasis. Peripheral massive infiltration of the tumor by neutrophils and eosinophils cells. Hematoxylin-eosin, magnification 20×. **H.** Adenocarcinoma, liver metastasis. Small nests of tumor cells in the liver parenchyma with low grade lymphohistiocytic infiltration. Hematoxylin-eosin, magnification 20×.

Moreover, we performed a comprehensive histopathological analysis of primary and metastatic tumors recovered 30 days PTI (Figure 4). All primary and metastatic tumors were identified as adenocarcinomas. These tumors were all highly cellular; neoplastic cells had a round-oval shape, large nuclei with vescicular chromatin and prominent, often multiple, nucleoli. Cells were anaplastic with marked anisocytosis and anisokaryosis and the mitotic index was always high (5 and more high-power field). The neoplastic cells were in clusters, separated by vascular or fibrovascular stroma. Necrotic foci were rare. Cells in the metastases in the liver and lungs resembled those in the primary tumors. Inflammation was present in all of the tumors from all groups. The inflammatory response was considerably more pronounced in STM-treated tumor-bearing mice compared to the untreated ones. The inflammation in untreated animals was mainly lymphocytic. By contrast, the inflammatory response in treated animals varied from lymphohistiocytic to neutrophilic/eosinophilic. Inflammatory cells were mainly seen at the periphery of the primary masses, but were disseminated throughout the metastases.

### STM induces a change in the subpopulation of immune cells in the spleen of treated mice

**Figure 5:**
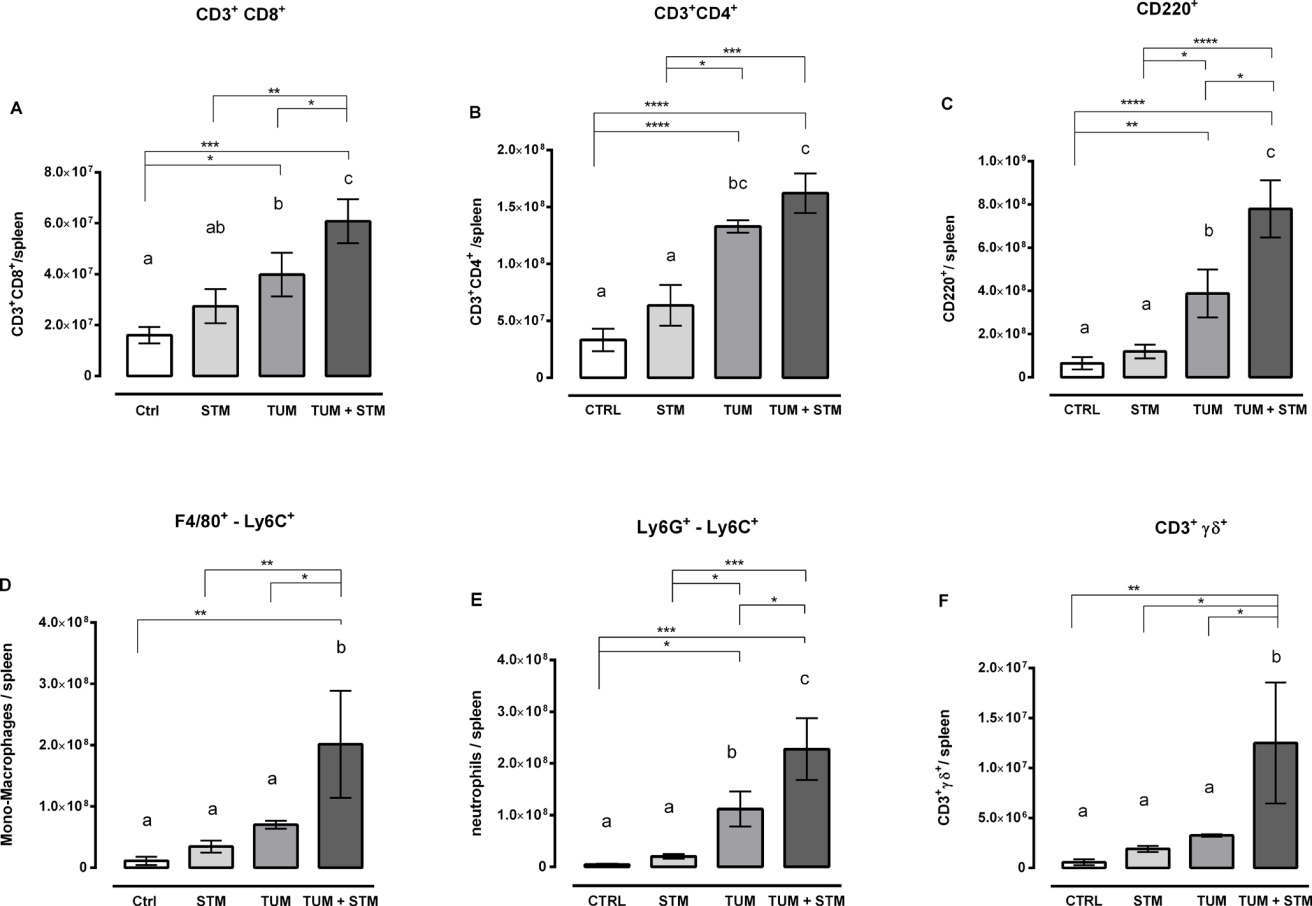
A-F. STM treatment modifies the phenotype of immune cells in the spleen The absolute number of CD4 and CD8 T cells, B cells, neutrophils, monocytes/macrophages and γδ T cells was analysed 30 days PTI, from 5 mice per group (one-way Anova Turkey's multiple comparison tests, data from one representative experiment out of two with similar results).

To further evaluate the effects of STM treatments, we determined, in a separate set of experiments, the modification occurring in the absolute number of monocytes/macrophages (F4/80^+^-Ly6C^+^), neutrophils (Ly6G^+^-Ly6C^+^), CD3^+^CD4^+^ T cells, CD3^+^CD8^+^ T cells, and B cells (CD220^+^) in spleens (Figure 5), as an indicator of the systemic environment. STM treatment induced a significant increase in the absolute numbers of monocytes/macrophages and neutrophils in spleens (Figure 5D and 5E). The absolute numbers of CD8^+^ T cells and B220^+^ cells were also higher in the spleen of STM-treated tumor-bearing mice when compared to controls (Figure 5A and 5C). On the other hand, the absolute number of CD4^+^ T cells was slightly increased but not statistically significant (Figure 5B).

Moreover, further analysis revealed an important increase in the number of double negative T cells (CD3^+^CD4^−^CD8^−^) in the spleens of STM-treated tumor-bearing mice compared to the control group. Therefore, we investigated the nature of these cells identifying in CD3^+^γδ^+^ T cells their prominent phenotype, significantly increased in STM-treated tumor-bearing mice compared to the control group (Figure 5F).

Taken together, these data suggest that monocytes/macrophages, neutrophils, CD8^+^ T cells, B220^+^ cells and CD3^+^γδ^+^ T cells were expanded in spleens of tumor-bearing mice in response to STM compared to control groups. An increase in the number and proportion of lymphocytes and in particular the expansion of granulocyte/monocyte compartment in the STM-treated group was confirmed through histopathological analysis (Figure 4).

### STM treatment induces a release of calreticulin *in vivo*

In searching for a candidate mechanism of action, our attention was focused on the possible role of calreticulin (CRT), a protein involved in the immunogenic cell death (ICD) pathway [26]. ICD is a particular form of cell death that involves changes in the composition of the cell surface as well as the release of soluble mediators, occurring in a defined temporal sequence. Since it has been known that in response to multiple ICD inducers a fraction of CRT translocate from the lumen of the endoplasmic reticulum to the surface of stressed and dying cancer cells [27, 28], we investigated the involvement of CRT in response to STM during *in vivo* treatments.

**Figure 6:**
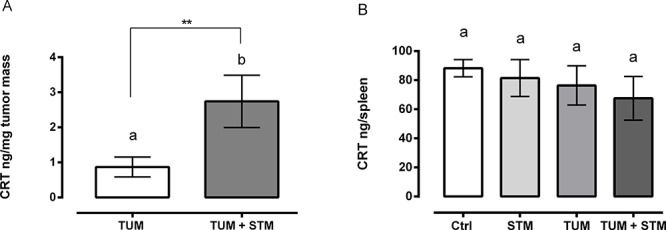
STM treatment induces a release of CRT in mice at 30 days PTI **A.** Production of CRT per mg of tumor mass in STM-treated tumor-bearing compared with untreated group (Mann Whitney unpaired *t*-test). **B.** Production of CRT in spleens of untreated and treated tumor-free and tumor-bearing mice (Uncorrected Fisher's LSD multiple comparison one-way ANOVA).

Therefore, we evaluated the amount of CRT in spleens and tumor masses in mice treated with the therapeutic administration (group 1), 10 and 30 days PTI. The results showed that, at 10 days PTI, there were no differences of CRT amount in tumor masses of both STM treated and untreated groups (Supplementary Figure S5). However, a significant increase of CRT per mg of tumor mass was observed at 30 days PTI in the STM-treated tumor-bearing mice, compared to the untreated tumor-bearing ones (Figure 6A). The total amount of CRT in the spleens in both untreated and treated tumor-bearing mice, was similar at day 10 and 30 PTI, underlining the concept that the CRT production induced by STM is localized to the tumor mass (Figure 6B and Supplementary Figure S5).

### STM treatment promotes IFN-γ and IL-1β induction in the tumor mass

**Figure 7:**
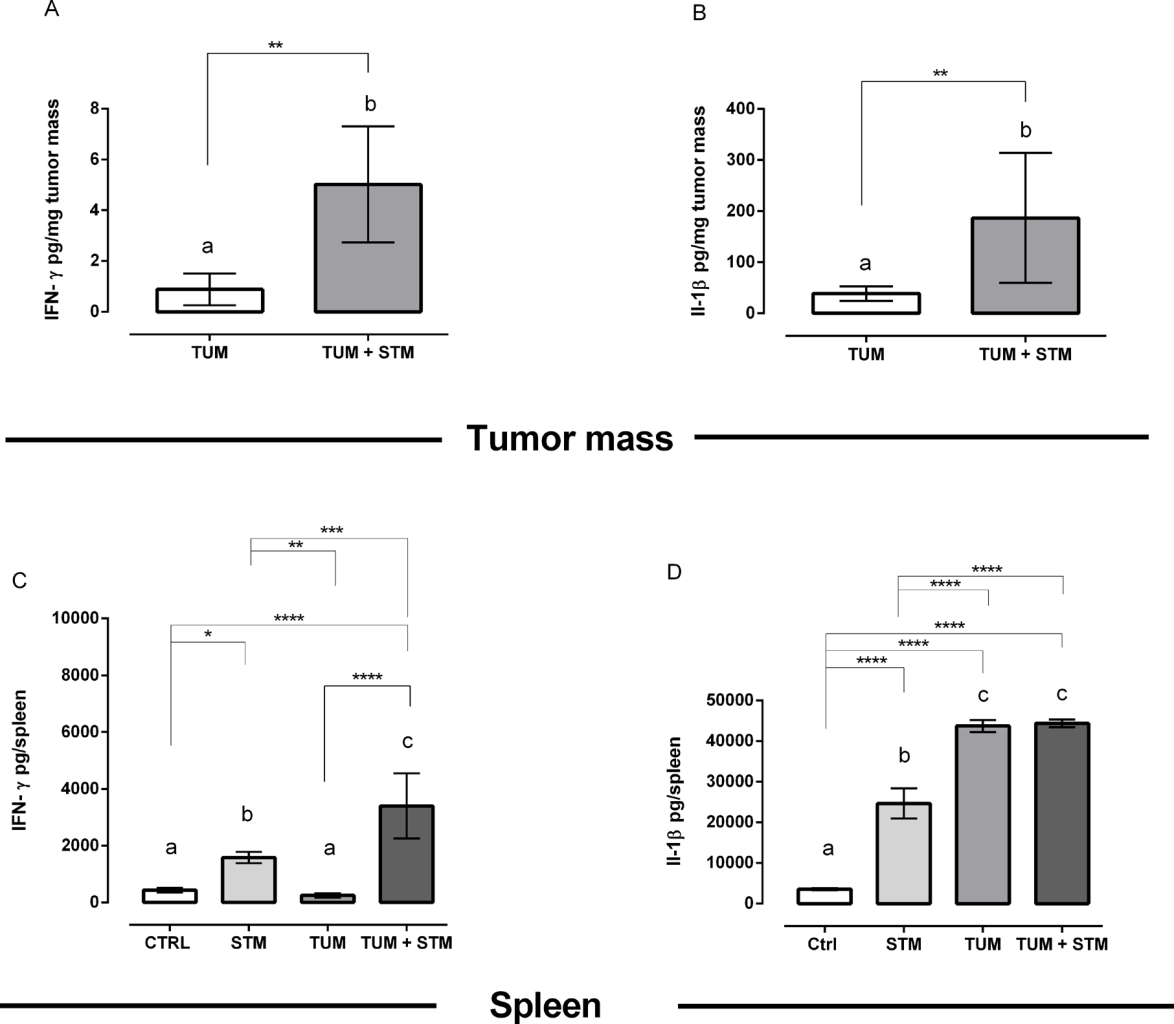
STM treatment promotes IFN-γ and IL-1β production in the tumor masses and spleens at 30 days PTI **A** and **B.** IFN-γ and IL-1β production, per mg of tumor masses, from mice treated with STM 30 days PTI (Mann Whitney unpaired *t*-test). **C** and **D.** Total amount of IFN-γ and IL-1β in the spleen of STM-treated and untreated tumor-bearing mice (Uncorrected Fisher's LSD multiple comparison one-way ANOVA).

We also evaluated the IFN-γ and IL-1β production in tumor masses and spleens at different times in both STM-treated and untreated groups. The concentration of cytokines was assessed at day 10 and 30 PTI (Figure 7A-7D and Supplementary Figure S6A–S6D). As shown in Figure 7A and 7B, tumor masses from mice treated with STM showed a significantly increased production, per mg of masses, of both IFN-γ and IL-1β cytokines at 30 days PTI (*P* < 0.01; Figure 7), whereas the IL-1β was increased already at 10 days PTI (*P* < 0.01, Supplementary Figure S6). Moreover, the total amount of IFN-γ in the spleens resulted significantly increased in STM-treated tumor-bearing mice at 30 days PTI (*P* < 0.01, Figure 7C) compared to the untreated tumor-bearing mice. The total amount of IL-1β in the spleens, instead, was comparable in both STM-treated and untreated tumor-bearing mice, at day 10 and 30 PTI, and significantly increased in comparison to free-tumor mice (Figure 7D and Supplementary Figure S6). These results indicate that the differences among groups were due to the tumor environment and not to the treatment with STM.

## DISCUSSION

In this work we investigated a therapy based on repeated SC administration of a live attenuated S. Typhimurium strain, herein indicated as STM, to treat a mouse model of mammary adenocarcinoma. Bacteria belonging to the *Salmonella* genus are facultative anaerobes that can grow in hypoxic or necrotic tumor areas. Auxotrophic mutant strains are expected to preferentially destroy tumor cells, which, as opposed to normal ones, are rich in free nutrients required for their replication [29]. Literature data suggest that some cancerous tissues (e.g.: lung, liver, kidney, breast) exhibit higher zinc concentrations than normal ones. Indeed, while zinc levels in serum of tumor-suffering patients are decreased, the concentration of zinc in cancer tissues is higher than in normal tissues [30]. Therefore, it is possible to expect that STM could selectively proliferate in such environments and finds a growth advantage in tumor mass.

We initially proved, through *in vitro* infection, the ability of STM to penetrate and induce tumor cell cytotoxicity in mammary adenocarcinoma 4T1 cells, as seen by a 40% reduction of viable cells in culture as compared to untreated cells (Figure 1A and 1B). These results were confirmed in other tumor cell lines, such as SiHa and TC1, suggesting that STM is able to penetrate in tumors of different embryological and animal origin. Successively, we provided evidence that SC therapeutic administration of STM elicits strong delay in tumor growth associated with an extended survival in tumor-bearing mice (Figure 3), and an important reduction in the frequency of metastases (Supplementary Figure S4). Finally, the STM treatment resulted in an important accumulation of bacteria, in particular in tumor sites (Supplementary Figure S2A and Figure 3C), which induces a potent inflammatory response, recruiting a large number of innate and adaptive immune cells in both spleens and tumor masses. In particular, the STM treatment induces a significant increase in the recruitment of CD8, B cells, monocytes/macrophages and neutrophils in spleen (Figure 5). The modifications in the immune populations were also evident in tumor site of mice treated with STM by histopathological analysis (Figure 4), which showed a marked inflammatory response that varied from lymphohistiocytic to neutrophilic/eosinophilic populations.

Antitumor effects of neutrophils, in particular after being activated by microorganism-derived products, were demonstrated in different tumor models [31–32]. Vendrell et al. recently reported that neutrophils are the main infiltrating tumor cells after treatment with S. Typhi, and hypothesized that neutrophils recruited by *Salmonella* can act as antigen-presenting cells (APC) expressing tumor antigens for priming of tumor-specific T cells [33]. Therefore, it is reasonable to hypothesize that sensing of *Salmonella* triggers neutrophil participation to control the infection. At the same time, in tumor bed, neutrophils can cause direct tissue damage due to their ability to produce reactive oxygen species and proteinases [34]. Moreover, they can alter the immune response through the production of cytokines and chemokines, which can recruit inflammatory cells, with a development of an adaptive anti-tumoral immune response, possibly favored by the presence of antigens derived from *Salmonella* invasion.

Besides that, we also found an increased numbers of CD3^+^ γδ^+^ T cells in spleens from tumor-bearing mice receiving the STM (Figure 5F). This finding is important because it is known that γδ T cells exhibit potent MHC-unrestricted lytic activity against different tumor cells *in vitro* and have been also consistently identified and isolated from tumor infiltrating lymphocytes in various types of cancer [35, 36]. Most of current immunotherapeutic approaches aim at inducing antitumor response stimulating the adaptive immune system, which is bound on MHC-restricted αβ T cells. However, the loss of MHC molecules is often observed in cancer cells, which makes tumor cells resistant to αβ T cell-mediated cytotoxicity [37, 38]. It implies that γδ^+^ T cells are actively involved in anti-cancer responses.

Concerning the possible involvement of an immune mediated killing pathway, particular attention was dedicated to the analysis of CRT release after treatment of tumor-bearing mice with STM. This resulted in a significantly increased level of CRT release from tumor masses, which conversely was not evident from the splenic cells (Figure 6). Zitvogel and Kroemer have clearly shown that certain chemotherapeutic regimens are able to trigger cancer cell death while stimulating endogenous immune responses against the tumor [39–44]. Many chemotherapeutic agents can upregulate multiple surface molecules on tumor cells, making them more sensitive to immune mediated killing [45, 46] without inducing classic ICD [47–49]. In particular, as a result of *pre mortem* endoplasmic reticulum stress and autophagy, cancer cells responding to ICD inducers expose CRT on the outer leaflet of their plasma membrane at a pre-apoptotic stage, in addition to other proteins involved in apoptotic processes and secondary necrosis, such as ATP and HMGB1. This facilitates the recruitment of DCs into the tumor bed, the engulfment of tumor antigens by DCs, stimulated by CRT, and optimal antigen presentation to T cells. Altogether, these processes result in a potent IL-1ß- and IL-17-dependent, IFN-γ-mediated immune response involving both γδ T cells and CTLs, which can lead to the eradication of chemotherapy-resistant tumor cells [26]. In this context, STM can facilitate the recruitment and activation of mononuclear-phagocytes into the tumor bed, and the induction of CRT exposure, which can stimulate the engulfment of tumor antigens and enhance their antigen-presenting capacity.

We have also found that STM treatment induced a significant increase in IFN-γ production in tumor cells and splenocytes at 30 days PTI (Figure 7A and 7C). There is compelling scientific evidence of the important role of IFN-γ during antitumor activity of *Salmonella* treatment [50]. IFN-γ, indeed, has been associated with cytostatic and cytotoxic anti-tumoral functions and stimulates the detection and elimination of tumor cells [51]. Moreover, we found that IL-1β cytokine was significantly increased in tumor masses but not at the systemic level, thus resulting tightly correlated to the tumor site (Figure 7B and 7D). The IL-1ß increase is known to be indispensable for setting off a cascade of events, which culminates in the recruitment and functional maturation of both innate γδ T cells and tumor antigen-specific CD8^+^ aß T lymphocytes [52].

In this picture, STM can trigger a pathway, which involves the activation of APCs, stimulating the immune response with production of proinflammatory cytokines and antigen cross-presentation to T-cells. Additionally, cancer cell death in a proinflammatory environment can trigger antitumor immunity by facilitating DCs tumor antigen cross-presentation to T-cells.

These data suggest that the anti-tumor effect of STM is the result of a combination of different mechanisms involving direct cytotoxicity on tumor cells and activation of the immune response resulting in a significant reduction of the tumor masses growth and in an increase of the life expectancy.

## MATERIALS AND METHODS

### Bacterial strain

Attenuated S. Typhimurium STM, corresponding to the previoulsly described strain SA186 (a znuABC deletion mutant of the S. Typhimurium strain ATCC 14028) [17, 21, 23], was cultured at 37°C in Brain Heart Infusion (Oxoid Ltd., UK), harvested by centrifugation and then washed twice in ice-cold Phosphate Buffer Solution (PBS) (Sigma-Aldrich, Milan, Italy).

### Animals and tumor cell lines

Female Balb/c mice, 6–8 weeks old were purchased from Charles River Laboratories (Charles River, Italy, SRL) and were used for *in vivo* experiments. All animal experiments were conducted according to current European legislation (Directive 2010/63/UE). Mouse mammary adenocarcinoma cell line 4T1 (ATCC: CRL-2539) were used for subcutaneous inoculation in Balb/c mice and *in vitro* assays.

Murine tumor cells TC1 cells (ATCC: CRL-2785, mouse lung tumor cells, co-transformed with human papillomavirus 16, E6/E7 and c-Ha-Ras) and human tumor cells SiHa (ATCC: HTB-35, grade II of human squamous cervix carcinoma cell) were employed for *in vitro* studies. All tumor cells were cultured in RPMI-1640 medium (Sigma-Aldrich, St. Louis, MO) supplemented with 10% fetal bovine serum (FBS, Gibco-BRL, USA), 2 mM L-glutamine, Gentamicin (100 μg/ml), at 37°C in 5% CO2 atmosphere.

### *In vitro* invasion and intracellular replication assays

4T1, TC1 and SiHa cells were seeded in 96-well plates to a density of 1 × 10^5^ cells per well. STM was diluted in a culture medium, added to the tumor cells at a multiplicity of infection of 100:1 and without bacteria, and incubated at 37°C in 5% CO_2_ atmosphere. After 1 h, cell cultures were rinsed and incubated in a medium containing gentamycin sulfate (100 μg/ml) to kill extracellular bacteria. Viable intracellular bacteria were recovered by lysing the cells in distilled water with 0.1% of Triton X-100 for 10 min. Plating serial dilutions on LBA plates performed quantification of bacteria. For intracellular replication assay, intracellular *Salmonella* were quantified at 2, 4 and 24 h.

### *Salmonella* cytotoxicity assays

*Salmonella* cytotoxicity on 4T1 cells was measured by a MTT cytotoxic assay, seeding in 96-well plates to a density of 1 × 10^5^ cells per well in 200 μl of RPMI with 10% FBS. STM was diluted in RPMI, added to the tumor cells at a MOI of 100:1 and incubated at 37°C in 5% CO2. After 1 h, the cell cultures were rinsed and incubated in a medium containing gentamycin sulfate (100 μg/ml) and incubated for 24 h. 4T1 cells were then re-suspended in 100 μl/well of complete RPMI, 10 μl/well of MTT solution (TACS MTT Cell Proliferation Assay, TREVIGEN, MD) was added and incubated for 3 h. 100 μl of detergent reagent were added and after 3 h the absorbance at 570 nm were measured.

### Evaluation of the antitumor effect of S*almonella in vivo*

4T1 cells were harvested, washed and suspended to a concentration of 5 × 10^4^ cells/ml in saline. Mice were injected SC into the right flank with 1 × 10^3^/100 μl cells and were monitored for tumor growth. STM was SC co-administrated or administrated PTI at 1 × 10^4^ cfu/100 μl. The tumor progression was monitored at different time points through ultrasound analysis (at day 14) and size measurements using-Vernier calipers (every 5 days), and an estimate of the tumor volumes was calculated.

### STM colonization

At designated times, the animals were sacrificed and tumors and spleens were removed and weighted. All tissues were homogenized in PBS and the number of viable STM was determined in each tissue by plating serial dilutions on LB agar plates. Data is presented as per mg of tissue.

### Histological analysis

The samples (5 animals per group) were taken at 30 days PTI, fixed in 10% neutral buffered formalin, processed according to routine histological methods, embedded in paraffin, and then sectioned at about 3–5 microns. The sections were then deparaffinized, rehydrated after immersion in a series of decreasing concentrations of alcohols and subsequently stained with hematoxylin-eosin and then examined under a light microscope. All primary and metastatic tumors from every mouse were examined histologically.

### Ultrasound analysis

All mice were analysed 14 days after the first co-administration of STM and 4T1 cells, and the total volume of tumor mass was assessed measuring the length of the principal axes. The limits of the nodule margins were evaluated picture clip photo clip. (GE LOGIQ e Ultrasound transducers, 12L-RS probe).

### Flow cytometry analysis of immune cells of tumor-bearing mice spleens

Analysis of immunological spleen-cells of mice treated or not with STM was conducted by flow cytometry 30 days PTI. The erythrocytes were lysed using lysis buffer before staining. Cells were immunostained with the following antibodies: FITC-CD3, PE-Cy7-CD8, PE-CD4, PE-Cy7-CD220, FITC-F4/80, FITC-ly6G, PE-Cy7-Ly6C, Pe-γδ (BD Pharmingen, San Diego, USA). Cells were harvested, washed with PBS-5% FBS and stained with antibodies for 15 min in the dark at 4°C. Cells were fixed for 20 min with 1% paraformaldehyde/PBS. A gate was set within the lymphocyte-monocyte populations in the FSC–SSC dot-plot and 50, 000 events were collected.

### ELISA

The concentrations of CRT, IL-1β and IFN-γ were quantified in mouse cell lysate from spleens and tumor masses using respective ELISA kits (Mouse Calreticulin ELISA kit, Cusabio Biotech CO, LTD; Mouse IL-1β/IL-1F2 and IFN-γ R&D Systems Europe, Ltd.) according to the manufacturer's instructions.

### Statistical analysis

Differences in survival times were determined using mortality curves (log-rank mantel-cox test). For the *in vitro* and *ex vivo* assays, the statistical significance of differences between study groups was analyzed using ANOVA and Student's *T* test; *p* < 0.05 was considered as statistically significant. These symbols were used to indicate the statistical significance: *P < 0.1, **P < 0.01, ***P < 0.001, ****P < 0.0001. Significant differences between two bars were marked with different letters.

## SUPPLEMENTARY FIGURES


