# Supplementary material for: Attenuated mutant strain of Salmonella Typhimurium lacking the ZnuABC transporter contrasts tumor growth promoting anti-cancer immune response
Source: Oncotarget. 2015 May 7;6(19):17648–60. doi: 10.18632/oncotarget.3893 (PMC4627335; doi:10.18632/oncotarget.3893)
Supplement: Supplementary file 1 [file oncotarget-06-17648-s001.pdf]

SUPPLEMENTARY FIGURES

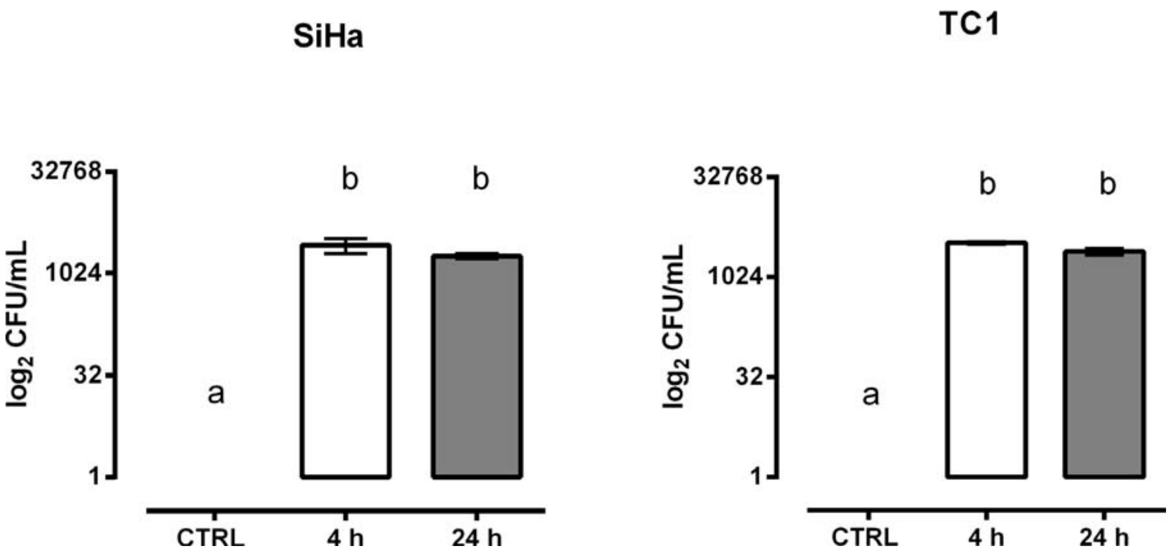

Supplementary Figure S1: STM colonizes cells of different embryologic nature and animal species. Intracellular colonization of STM into SiHa and TC1 tumor cells at 4 and 24 h post-treatment (Mann-Whitney unpaired *t*-test).

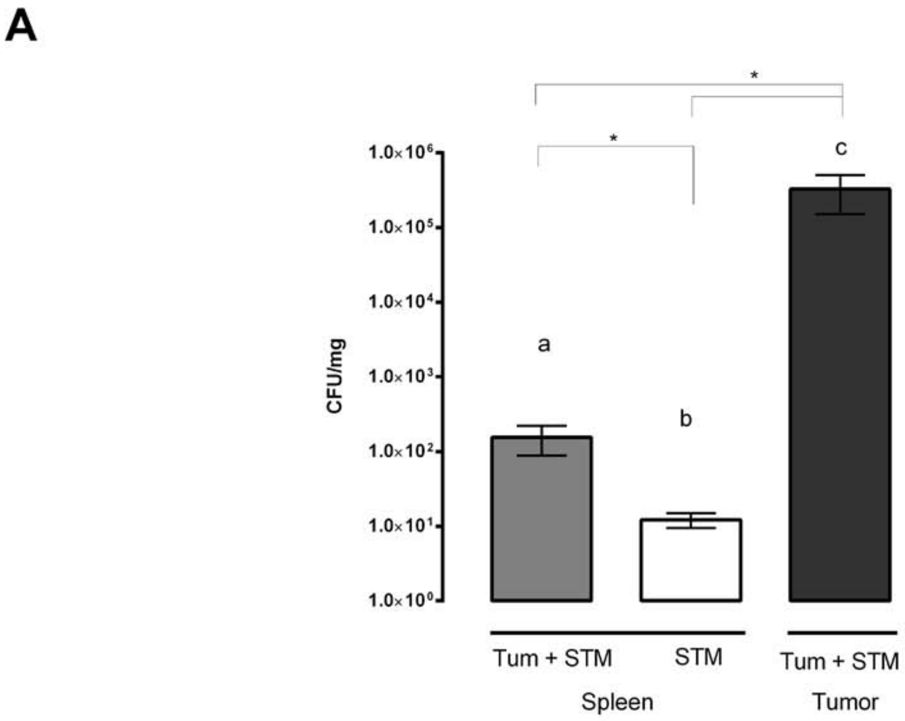

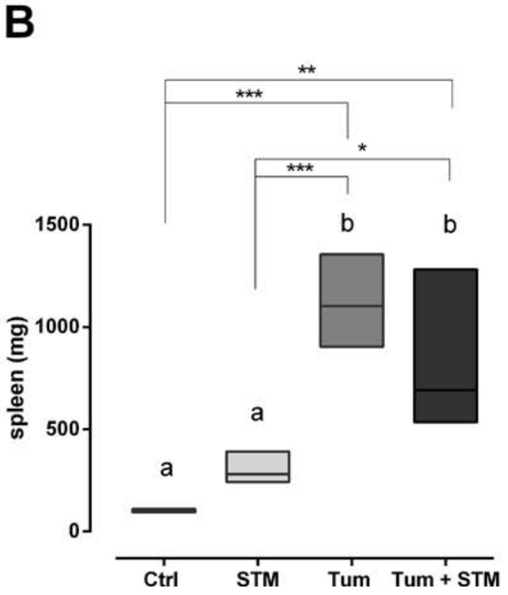

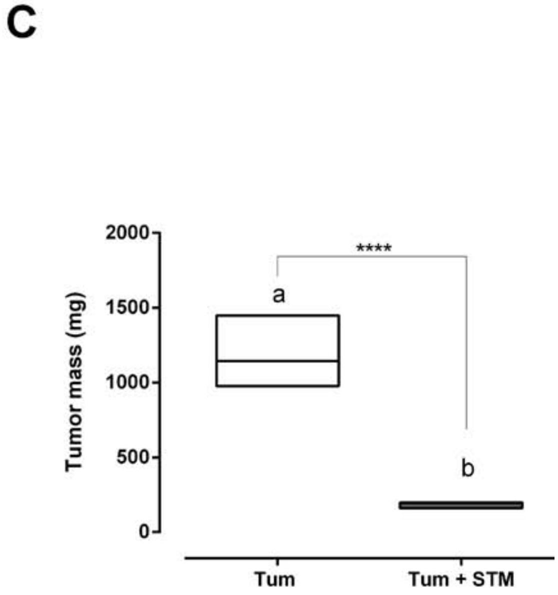

Supplementary Figure S2: Number of bacteria in tumor masses and spleens in co-administration treatment after 30 days. A. Mice were sacrificed 30 days after treatment and the number of bacteria in tumor and spleen was determined. Graphs show mean of CFU per mg of tissue (from 5 animals, one-way Anova Turkey's multiple comparisons test). **B and C.** Weight of spleens and tumor masses of STM-treated and untreated tumor-bearing mice (*t*-test analysis).

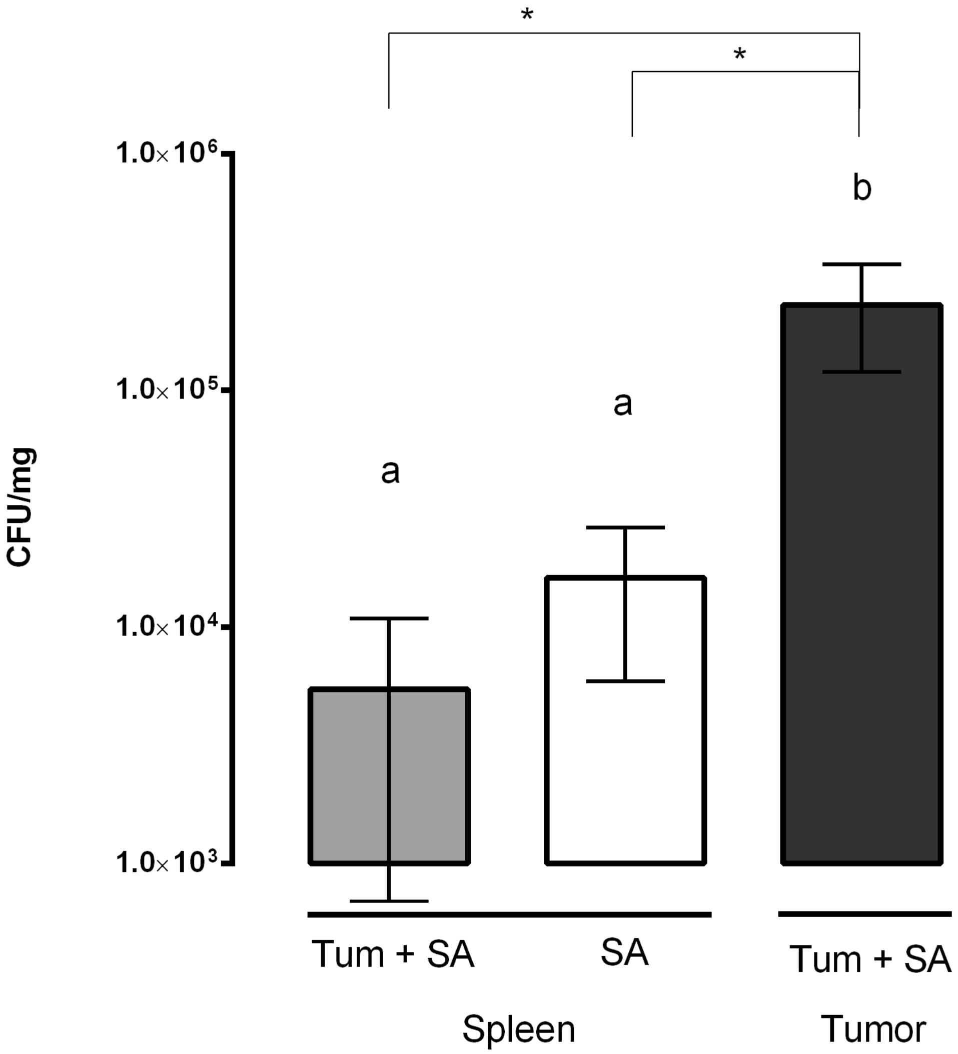

Supplementary Figure S3: Number of bacteria in tumors and spleens in therapeutic administration protocol 10 days PTI. Tumor-bearing and free-tumor mice both treated with STM were sacrificed at days 10 PTI and the number of bacteria in tumor and spleen was determined. Graphs show mean of CFU per mg of tissue (6 animals per group, Mann-Whitney unpaired *t*-test).

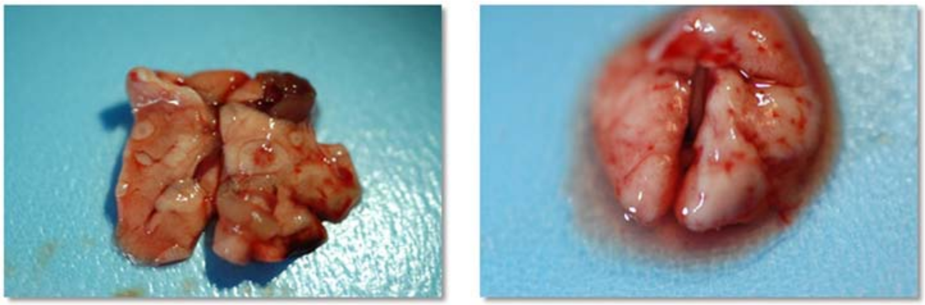

Supplementary Figure S4: STM inhibits the metastatic properties of the tumor cells. The lungs were removed from mice 30 days PTI. **Left panel**: untreated group has a compromised lung tissue with the presence of lung tumor nodules. **Right panel**: STM-treated group presents a dramatic decrease in the incidence of the metastases with tumor-free lung tissue. The pictures are representative experiment of one mouse out of ten with similar results.

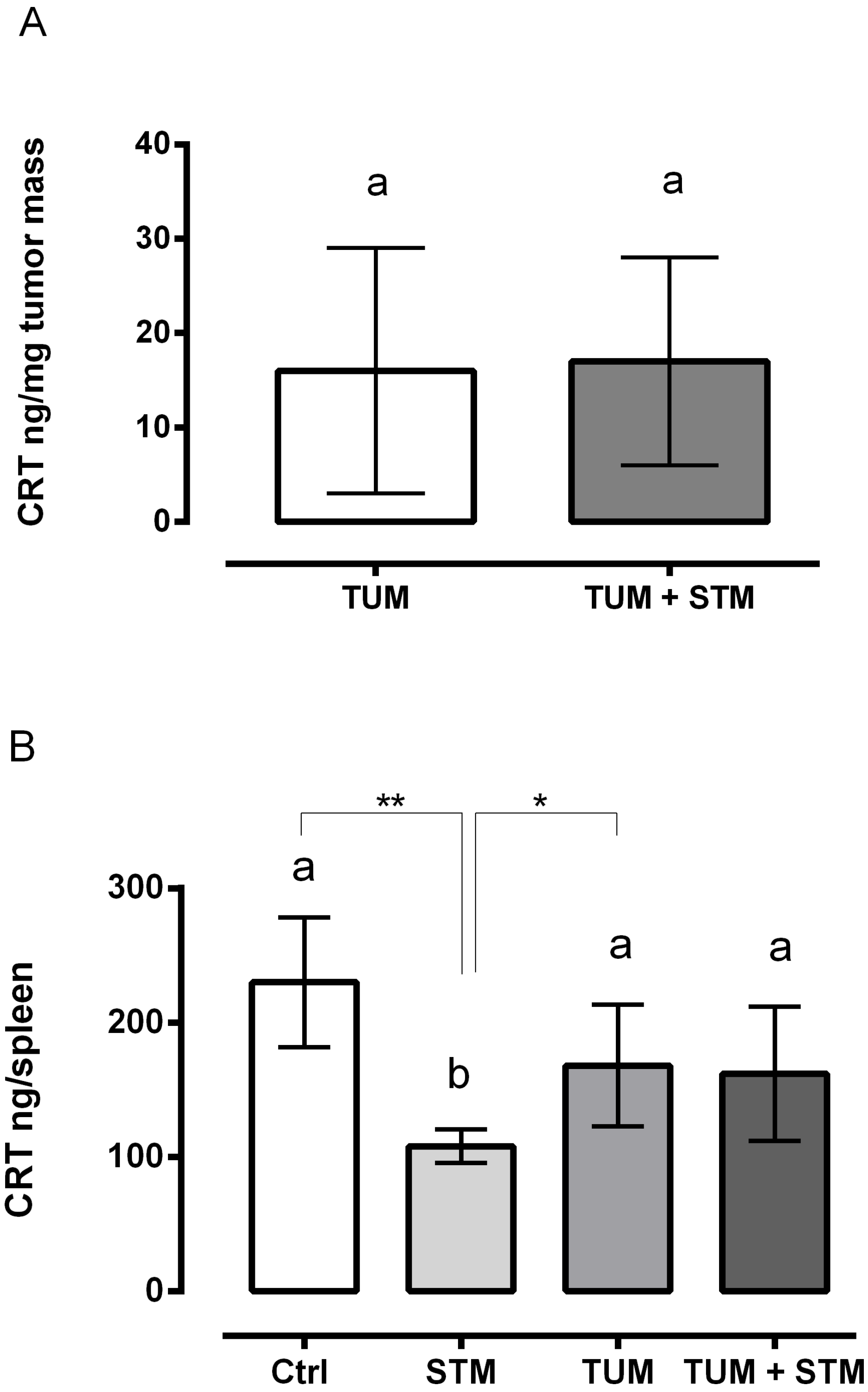

Supplementary Figure S5: Release of Calreticulin after STM treatment in mice after 10 days PTI. A. Production of CRT per mg of tumor mass in STM-treated tumor-bearing compared with untreated group (Mann Whitney unpaired *t*-test). **B.** Production of CRT in spleens of untreated and treated tumor-free and tumor-bearing mice (Uncorrected Fisher's LSD multiple comparison one-way ANOVA).

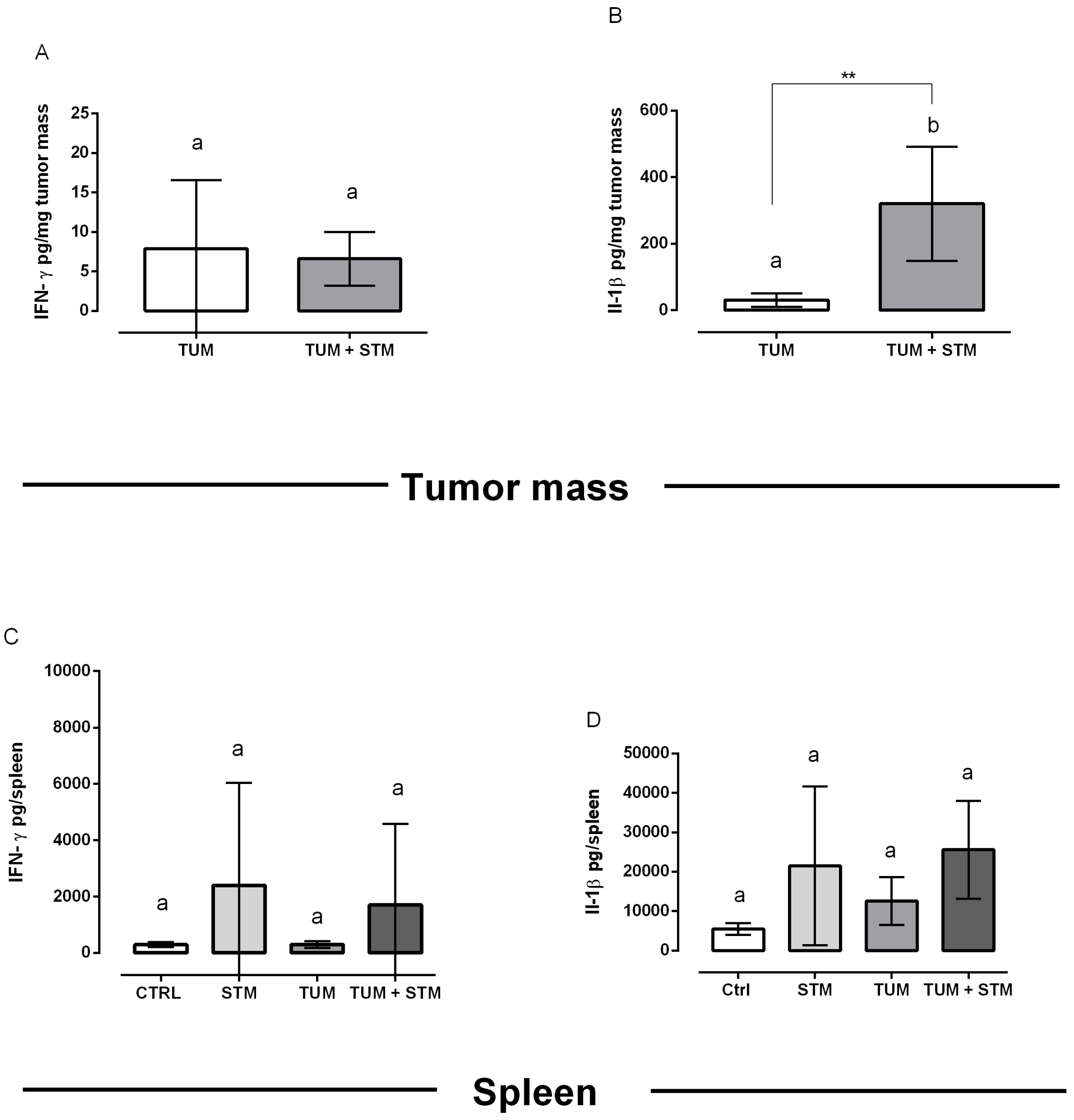

Supplementary Figure S6: IFN-γ and IL-1β production in the tumor masses and spleens after 10 days PTI. A and B. IFN-γ and IL-1β production, per mg of tumor masses, from mice treated with STM (Mann Whitney unpaired *t*-test). **C and D.** Total amount of IFN-γ and IL-1β in the spleen of STM-treated and untreated tumor-bearing mice (Uncorrected Fisher's LSD multiple comparison one-way ANOVA).
